# Factors Creating a Need for Repeated Drainage of Deep Neck Infections

**DOI:** 10.3390/diagnostics12040940

**Published:** 2022-04-09

**Authors:** Chia-Ying Ho, Yu-Chien Wang, Shy-Chyi Chin, Shih-Lung Chen

**Affiliations:** 1 Division of Chinese Internal Medicine, Center for Traditional Chinese Medicine, Chang Gung Memorial Hospital, Taoyuan 333, Taiwan; chiayingho23@gmail.com; 2 School of Medicine, Chang Gung University, Taoyuan 333, Taiwan; m7054@cgmh.org.tw (Y.-C.W.); b25chin@gmail.com (S.-C.C.); 3 Department of Otorhinolaryngology & Head and Neck Surgery, New Taipei Municipal TuCheng Hospital (Built and Operated by Chang Gung Medical Foundation), New Taipei City 236, Taiwan; 4 Department of Otorhinolaryngology & Head and Neck Surgery, Chang Gung Memorial Hospital, Linkou 333, Taiwan; 5 Department of Medical Imaging and Intervention, Chang Gung Memorial Hospital, Linkou 333, Taiwan

**Keywords:** blood sugar, deep neck infection, incision and drainage, multiple space involvement, re-operation

## Abstract

Deep neck infection (DNI) is associated with morbidity and mortality. Surgical incision and drainage (I&D) of DNI abscesses are essential. Refractory abscesses require repeat I&D. Few studies have assessed the risk factors associated with repeat I&D; here, we investigated such factors. In total, 605 patients with DNI were enrolled between July 2016 and February 2022. Of these patients, 107 underwent repeat I&D. Clinical variables were assessed. On univariate analysis, a high blood sugar level (odds ratio (OR) = 1.006, *p* < 0.001), the involvement of at least four neck spaces (OR = 15.44, *p* < 0.001), and mediastinitis (OR = 1.787, *p* = 0.040) were significant risk factors for repeat I&D. On multivariate analysis, a high blood sugar level (OR = 1.005, *p* < 0.001) and the involvement of at least four neck spaces (OR = 14.79, *p* < 0.001) were significant independent risk factors for repeat I&D. Patients who required repeat I&D had longer hospital stays and a higher tracheostomy rate than did other patients (both *p* < 0.05). The pathogens did not differ between patients who did and did not require repeat surgical I&D (all *p* > 0.05), but the rates of pathogen non-growth from blood cultures were 19.47% (97/498) in the group without a need for repeat I&D and 0.93% (1/107) in the group with such a need (*p* < 0.001). DNI can be fatal; a higher blood sugar level and the involvement of at least four neck spaces were independent risk factors for repeat surgical I&D. If at least four neck spaces are involved, we recommend controlling the blood sugar level after admission. We found significant differences in the length of hospital stay and the need for tracheostomy between groups who did and did not require repeat surgical I&D. Although the pathogens did not differ between the groups, pathogen non-growth from blood cultures was less common in the group with for repeat surgical I&D than in the group without such a need.

## 1. Introduction

Deep neck infection (DNI) is a life-threatening bacterial infection [1]. Clinical manifestations of DNI include neck swelling and redness, localized heat, and shortness of breath; some patients exhibit airway obstruction. DNI can trigger severe sepsis, esophageal perforation, necrotizing fasciitis, descending necrotizing mediastinitis, disseminated intravascular coagulation, jugular vein thrombosis, and pericarditis [2,3,4,5,6,7,8,9]. In addition to antibiotics, surgical drainage and postoperative wound irrigation are required by patients with advanced DNI [10].

Surgery requires great care; the anatomical landmarks of DNI patients are not clearly evident [11,12]. Because major blood vessels such as the carotid artery and the jugular vein are located in the cervical region, precise abscess drainage is difficult [13]. Repeat surgery is required by some patients during the hospital stay [14,15]. Therapeutic management is complicated. However, few studies have sought risk factors associated with repeat surgical drainage; here, we investigated such factors.

## 2. Materials and Methods

**Figure 1:**
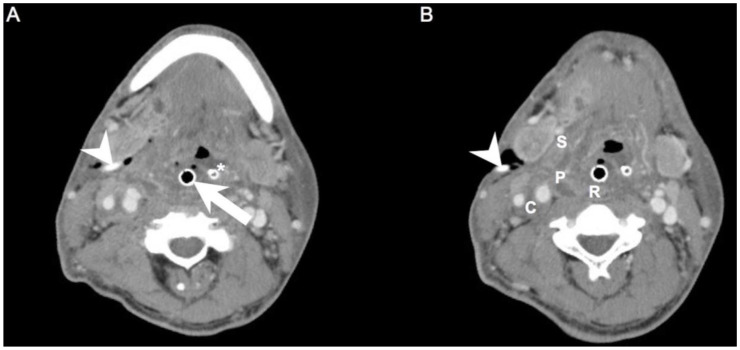
(**A**,**B**). Axial views of a patient who required repeat I&D. Arrowhead: drainage tube of the first surgery; Arrow: endotracheal tube; Asterisk: nasogastric tube; C: carotid space; P: parapharyngeal space; R: retropharyngeal space; S: submandibular space.

We retrospectively reviewed the medical records of 605 patients with DNIs who were admitted to Chang Gung Memorial Hospital (Linkou, Taiwan) between July 2016 and February 2022. DNI was diagnosed by ultrasonography and computed tomography (CT) [7]. The involved deep neck spaces of CT images were identified retrospectively by at least one radiologist (S.-C.C.) and one otorhinolaryngologist (S.-L.C.). If there were situations where the involved deep neck spaces were difficult to identify, it would be decided by team discussion. Treatment included antibiotics and surgical incision and drainage (I&D). I&D was performed when the DNI compromised the airway, the abscess was large (≥2 cm), and when the DNI did not improve after 48 h of intravenous empirical antibiotics [16]. These antibiotics were ceftriaxone 1 g/12 h and metronidazole 500 mg/8 h; they reduced the levels of aerobic and anaerobic bacteria before culture results were available [17,18]. Postoperative irrigation was performed at intervals of 4–8 h depending on disease severity [19]. Repeat I&D was defined as more than one drainage procedure during the same hospital stay. We followed the patient’s clinical condition and laboratory data after the first open I&D. If the patient’s symptoms and data progressed seriously within 48–72 h, we further arranged a CT exam. If there were abscesses that must be drained, the clinician would arrange a repeated open I&D (Figure 1A,B).

### 2.1. Exclusion Criteria

The exclusion criteria were a swallowed foreign body, severe cardiopulmonary disease, previous head-and-neck tumor surgery, and prior head-and-neck chemoradiotherapy. Follow-up CT was used during hospitalization to exclude patients whose abscesses were initially ineffectively drained. In total, 605 patients were enrolled, of whom 107 required repeat drainage.

### 2.2. Data Collection

We recorded gender, age, C-reactive protein (CRP) and blood sugar levels, diabetes mellitus (DM) status, number of involved DNI spaces, mediastinitis status, length of hospital stay, tracheostomy status, all I&D procedures, and pathogens involved.

### 2.3. Statistical Analysis

All data were analyzed using MedCalc software (ver. 18.6; MedCalc, Ostend, Belgium). Because the Kolmogorov–Smirnov test showed that the data were not normally distributed, we used the chi-squared test to compare categorical variables and the Mann–Whitney *U* test to compare continuous variables. We employed logistic regression for univariate and multivariate analyses. We engaged in forward stepwise selection, which was followed by multivariate logistic regression; all variables included in univariate analysis were entered into the final multivariate model. For all analyses, *p* < 0.05 was considered to indicate statistical significance.

## 3. Results

Demographic and clinical data are listed in Table 1. In total, 605 DNI patients (397 men (65.62%) and 208 women (34.38%); mean age, 51.70 ± 18.66 years) were included. The mean CRP level was 147.96 ± 107.74 mg/L and the mean blood sugar level was 152.52 ± 79.34 mg/dL. Overall, 254 (41.98%) patients had DM. Involvement in all patients comprised one deep neck space in 153 (25.28%) patients, two spaces in 183 (30.24%) patients, three spaces in 156 (25.78%) patients, and at least four spaces in 113 (18.70%) patients.

Of those with deep neck space involvement, it involved the parapharyngeal space in 342 (56.52%) patients, the submandibular space in 243 (40.16%), the retropharyngeal space in 213 (35.20%), the masticator space in 124 (20.49%), the parotid space in 117 (19.33%), the carotid space in 58 (9.58%), the anterior cervical space in 57 (9.42%), the perivertebral space in 54 (8.92%), the visceral space in 53 (8.77%), and the posterior cervical space in 21 (3.47%). Mediastinitis was apparent in 85 (14.04%) patients. The mean length of hospital stay was 11.56 ± 9.18 days. Tracheostomy was performed on 120 (19.83%) patients. In total, 269 (44.46%) patients underwent I&D; 107 (17.68%) patients underwent at least two I&D procedures. Table 1 lists the cultured pathogens. The overall non-growth pathogen rate was 16.19% (98/605). 

Table 2 shows the results of univariate analyses. A high blood sugar level (odds ratio (OR) = 1.006, 95% confidence interval (CI): 1.003–1.008, *p* < 0.001), the involvement of at least four spaces (OR = 15.44, 95% CI: 9.443–25.26, *p* < 0.001), and mediastinitis (OR = 1.787, 95% CI: 1.043–3.061, *p* = 0.040) were significant risk factors for repeat I&D. All factors were subjected to forward stepwise selection, which was followed by multivariate logistic regression. A high blood sugar level (OR = 1.005, 95% CI: 1.003–1.008, *p* < 0.001) and the involvement of at least four spaces (OR = 14.79, 95% CI: 8.931–24.49, *p* < 0.001) were significant independent risk factors for repeat I&D (Table 2).

Table 3 lists the hospital stays and tracheostomy statuses of the 107 patients who underwent repeat I&D and the 498 patients who did not. The hospital stay was significantly longer for patients who underwent repeat I&D (18.19 ± 9.06 days) than for patients who did not undergo repeat I&D (10.13 ± 8.56 days, *p* < 0.001). The tracheostomy rate was significantly higher for patients who underwent repeat I&D (66.35%, 71/107) than for patients who did not undergo repeat I&D (9.83%, 49/498).

Table 4 lists the pathogens in both groups; there were no significant differences (all *p* > 0.05). However, no pathogens grew from blood cultures for 0.93% (1/107) of patients who underwent repeat I&D and for 19.47% (97/498) of patients who did not require repeat I&D; this difference was statistically significant (*p* < 0.001).

## 4. Discussion

DNI can trigger life-threatening complications; management includes broad-spectrum intravenous antibiotics, timely surgical drainage, and airway protection (i.e., intubation and/or tracheostomy) [20]. The annual incidence of DNI is approximately 9–15/100,000; the most common etiology is dental [21,22]. Obregon-Guerrero reported that of 87 patients with DNIs, 21 (24%) required re-operation [14]. This burdens the healthcare system and increases medical costs [15,23]. Our rate of repeat I&D was 17.68%. A higher blood sugar level and the involvement of at least four spaces were independent risk factors associated with repeat I&D. Patients with such factors required longer hospital stays and a higher rate of tracheostomy than did other patients. Although the pathogens did not differ between groups, pathogen non-growth from blood was less common in the repeat I&D group (0.93%, 1/107) than in the other group (19.47%, 97/498). As shown in Table 2, a higher blood sugar level, the involvement of at least four spaces, and mediastinitis were risk factors for repeat I&D on univariate analysis.

Mediastinitis can be fatal; DNI spreads along the cervical fascia and the neck spaces down to the mediastinum [24]. The mortality rate was reportedly near 40% prior to the 1990s [25]. As diagnostic methods, surgical techniques, and intensive care unit protocols improved over the past 20 years, the outcomes and mortality have also improved. Multidisciplinary approaches and comprehensive medical treatments have significantly reduced mortality [9]. We found that the mediastinitis rate did not differ between the two groups on multivariate analysis. Mediastinitis indicates a severe infection. However, mediastinitis is not necessarily associated with incomplete surgical drainage. If mediastinitis did not occur in a site that was difficult to drain or irrigate, repeat I&D would be unnecessary.

We found that a higher blood sugar level and DNI involving at least four spaces were independent risk factors for repeat I&D on both univariate and multivariate analyses. Liu et al. reported that a high preoperative blood sugar was independently correlated with persistent discharge after operation to treat DNI [26]. Moreover, early blood sugar control has been shown to reduce infectious complications [27,28]. Importantly, we found that DNI involving at least four spaces was an independent risk factor for repeat I&D. The involvement of multiple spaces indicates an advanced infection [1] and increases mortality; elderly patients tend to exhibit more involved spaces than do patients aged <18 years [29]. Multiple-space DNI is associated with severe inflammation and increases the volume of cryptic space that is difficult to irrigate. Although blood sugar level can be the underlying reason for the repeat I&D, it can also be the effect of a more serious inflammatory response due to infection or even sepsis. We still recommend controlling the blood sugar level, especially in DNI patients who exhibit involvement of multiple spaces.

Table 3 shows that the length of hospital stay and tracheostomy rate were higher in the repeat I&D group than in the other group. Careful airway protection with close surveillance of respiratory function and adequate infection control are important considerations [30]. Ideally, airway specialists (otolaryngologists and anesthesiologists) should evaluate all patients early to prepare for airway security. The involvement of multiple spaces was previously identified as a risk factor for tracheostomy [31]. Patients undergoing repeat I&D should be closely monitored until airway signs and symptoms resolve [32]. Postoperative ventilation for >48 h was independently associated with unplanned re-operation [15], which is consistent with the findings of other studies: prolonged ventilation was a risk factor for re-operation and re-admission [33,34]. Older age and DM were correlated with a longer hospital stay [35].

Table 4 shows that the pathogens did not significantly differ between groups. The rate of specific pathogen non-growth from blood cultures was 19.47% (97/498) in the group that did not undergo repeat I&D, while it was only 0.93% (1/107) in the group that did undergo this procedure, which differed significantly. In addition, the overall rate of specific pathogen non-growth was 16.19% (98/605). Blood culture does not sensitively identify pathogens, especially when antibiotics have been administered [36,37].

Limitations of the Article

This study has some limitations. First, the retrospective nature of the study at a single institution resulted in a certain attrition rate. Furthermore, the repeated drainage for DNI is clinical and determined by the clinician. As such, while the independent variables are logical for repeated drainage, they do not mandate the requirement for repeated drainage. The bias occurred in this mode of data acquisition.

## 5. Conclusions

A higher blood sugar level and the involvement of at least four spaces were independent risk factors for repeat I&D. In such patients, the blood sugar level should be controlled after admission. The repeat I&D group exhibited a longer hospital stay and a higher tracheostomy rate than did the other group. Although the pathogens did not differ between the two groups, specific pathogen non-growth from blood was less common in the repeat I&D group than in the other group.

## Figures and Tables

**Table 1:** Clinical characteristics of the 605 DNI patients undergoing surgical drainage.

Characteristics	N (%)
Gender	605 (100.00)
Male	397 (65.62)
Female	208 (34.38)
Age, years (SD)	51.70 ± 18.66
CRP, mg/L (SD)	147.96 ± 107.74
Blood sugar, mg/dL (SD)	152.52 ± 79.34
Diabetes mellitus	254 (41.98)
Number of deep neck space involvement	
Single space	153 (25.28)
Double spaces	183 (30.24)
Triple spaces	156 (25.78)
Multiple spaces, ≥4	113 (18.70)
Deep neck space involvement	
Parapharyngeal space	342 (56.52)
Submandibular space	243 (40.16)
Retropharyngeal space	213 (35.20)
Masticator space	124 (20.49)
Parotid space	117 (19.33)
Carotid space	58 (9.58)
Anterior cervical space	57 (9.42)
Perivertebral space	54 (8.92)
Visceral space	53 (8.77)
Posterior cervical space	21 (3.47)
Mediastinitis	85 (14.04)
Length of hospital stay, days (SD)	11.56 ± 9.18
Tracheostomy	120 (19.83)
Surgical I&D	269 (44.46)
Repeated surgical I&D, ≥2	107 (17.68)
Pathogens	
*Streptococcus constellatus*	122 (20.16)
*Prevotella intermedia*	76 (12.56)
*Parvimonas micra*	73 (12.06)
*Klebsiella pneumoniae*	70 (11.57)
*Streptococcus anginosus*	66 (10.90)
*Prevotella buccae*	65 (10.74)
*Staphylococcus aureus*	37 (6.11)
*Streptococcus salivarius*	28 (4.62)
*Streptococcus pneumoniae*	25 (4.13)
*Gemella morbillorum*	24 (3.96)
*Staphylococcus epidemidis*	22 (3.63)
*Serratia marcescens*	22 (3.63)
*Eikenella corrodens*	19 (3.14)
*Streptococcus oralis*	17 (2.80)
*Salmonella enterica*	16 (2.64)
*Pseudomonas aeruginosa*	15 (2.47)
*Slackia exigua*	13 (2.14)
*Rothia amarae*	11 (1.81)
*Stenotrophomonas maltophilia*	11 (1.81)
No growth	98 (16.19)

DNI = deep neck infection; N = numbers; SD = standard deviation; CRP = C-reactive protein (normal range <5 mg/L); Blood sugar (normal range: 70–100 mg/dL); Incision and drainage = I&D.

**Table 2:** Univariate and multivariate analyses of data concerning 107 patients who required repeat I&D and 498 patients who did not.

Variable	Repeated Surgical I&D		Univariate Analysis			Multivariate Analysis		
	Yes	No	OR	95% CI	*p*-Value	OR	95% CI	*p*-Value
Gender	107	498			0.875			
Male	71	326	1.040	0.643–1.555				
Female	36	172	1.000					
Age, years					0.912			
>60	40	189	0.976	0.634–1.502				
≤60	67	309	1.000					
CRP, mg/L (SD)	160.18 ± 106.54	145.34 ± 107.92	1.001	0.999–1.003	0.199			
Blood sugar, mg/dL (SD)	191.69 ± 91.45	144.10 ± 73.92	1.006	1.003–1.008	<0.001 *	1.005	1.003–1.008	<0.001 *
Diabetes mellitus					0.082			
Yes	53	201	1.450	0.953–2.205				
No	54	297	1.000					
Multiple spaces, ≥4					<0.001 *			<0.001 *
Yes	66	47	15.44	9.443–25.26		14.79	8.931–24.49	
No	41	451	1.000			1.000		
Parapharyngeal space					0.588			
Yes	63	279	1.123	0.654–1.527				
No	44	219	1.000					
Submandibular space					0.660			
Yes	45	198	1.099	0.719–1.679				
No	62	300	1.000					
Retropharyngeal space					0.293			
Yes	33	180	0.787	0.502–1.234				
No	74	318	1.000					
Masticator space					0.182			
Yes	27	97	1.395	0.855–2.276				
No	80	401	1.000					
Parotid space					0.724			
Yes	22	95	1.098	0.653–1.845				
No	85	403	1.000					
Carotid space					0.101			
Yes	15	43	1.725	0.919–3.236				
No	92	455	1.000					
Anterior cervical space					0.689			
Yes	9	48	0.861	0.408–1.813				
No	98	450	1.000					
Perivertebral space					0.214			
Yes	13	41	1.541	0.795–2.989				
No	94	457	1.000					
Visceral space					0.096			
Yes	14	39	1.771	0.924–3.394				
No	93	459	1.000					
Posterior cervical space					0.211			
Yes	6	15	1.912	0.724–5.049				
No	101	483	1.000					
Mediastinitis					0.040 *	-	-	-
Yes	22	63	1.787	1.043–3.061				
No	85	435	1.000					

DNI = deep neck infection; Incision and drainage = I&D; SD = standard deviation; OR = odds ratio; CI = confidence intervals; CRP = C-reactive protein; * *p* < 0.05 shown in bold.

**Table 3:** Hospital stay lengths and tracheostomy statuses concerning 107 patients who required repeat I&D and 498 patients who did not.

Characteristics	Repeated, N = 107 (%)	Non-Repeated, N = 498 (%)	*p*-Value
Length of hospital stay, days (SD)	18.19 ± 9.06	10.13 ± 8.56	<0.001 *
Tracheostomy			<0.001 *
Yes	71 (66.35)	49 (9.83)	
No	36 (33.65)	449 (90.17)	

I&D = incision and drainage; DNI = deep neck infection; N = number; * *p* < 0.05 shown in bold.

**Table 4:** Pathogens concerning 107 patients who required repeat I&D and 498 patients who did not.

Pathogens	Repeated, N = 107 (%)	Non-Repeated, N = 498 (%)	*p*-Value
*Streptococcus constellatus*	24 (22.42)	98 (19.67)	0.520
*Prevotella intermedia*	15 (14.01)	61 (12.24)	0.630
*Parvimonas micra*	11 (10.28)	62 (12.44)	0.511
*Klebsiella pneumoniae*	12 (11.21)	58 (11.64)	0.907
*Streptococcus anginosus*	9 (8.41)	57 (11.44)	0.346
*Prevotella buccae*	10 (9.34)	55 (11.04)	0.587
*Staphylococcus aureus*	9 (8.41)	28 (5.62)	0.296
*Streptococcus salivarius*	8 (7.47)	20 (4.01)	0.148
*Streptococcus pneumoniae*	2 (1.86)	23 (4.61)	0.156
*Gemella morbillorum*	2 (1.86)	22 (4.41)	0.179
*Staphylococcus epidemidis*	6 (5.60)	16 (3.21)	0.259
*Serratia marcescens*	4 (3.73)	18 (3.61)	0.950
*Eikenella corrodens*	4 (3.73)	15 (3.01)	0.704
*Streptococcus oralis*	2 (1.86)	15 (3.01)	0.491
*Salmonella enterica*	3 (2.80)	13 (2.61)	0.910
*Pseudomonas aeruginosa*	5 (4.67)	10 (2.00)	0.140
*Slackia exigua*	2 (1.86)	11 (2.20)	0.818
*Rothia amarae*	2 (1.86)	9 (1.80)	0.965
*Stenotrophomonas maltophilia*	4 (3.73)	7 (1.40)	0.136
No growth	1 (0.93)	97 (19.47)	<0.001 *

I&D = incision and drainage; DNI = deep neck infection; N = number. * *p* < 0.05 shown in bold.

## Data Availability

All data generated or analyzed during this study are included in this published article. The data are available on request.

## Abbreviations

CT	Computed tomography
CRP	C-reactive protein
DNI	Deep neck infection
DM	Diabetes mellitus
I&D	Incision and drainage
OR	Odds ratio
